# Method for calculating confidence intervals for phase contrast flow measurements

**DOI:** 10.1186/1532-429X-16-46

**Published:** 2014-06-24

**Authors:** Michael S Hansen, Laura J Olivieri, Kendall O’Brien, Russell R Cross, Souheil J Inati, Peter Kellman

**Affiliations:** 1 National Heart, Lung, and Blood Institute, National Institutes of Health, 9000 Rockville Pike, Bethesda, MD 20814, USA; 2 Division of Cardiology, Children’s National Medical Center, 111 Michigan Ave., N.W, Washington, DC 20010, USA; 3 National Institute of Mental Health, National Institutes of Health, 9000 Rockville Pike, Bethesda, MD 20814, USA

**Keywords:** Phase contrast, Flow, Confidence intervals, Standard deviation, Region of interest

## Abstract

**Background:**

Phase contrast (PC) measurements play an important role in several cardiovascular magnetic resonance (CMR) protocols but considerable variation is observed in such measurements. Part of this variation stems from the propagation of thermal noise from the measurement data through the image reconstruction to the region of interest analysis used in flow measurement, which limits the precision. The purpose of this study was to develop a method for direct estimation of the variation caused by thermal noise and to validate this method in phantom and in vivo data.

**Methods:**

The estimation of confidence intervals in flow measurements is complicated by noise correlation among the image pixels and cardiac phases. This correlation is caused by sequence and reconstruction parameters. A method for the calculation of the standard deviation of region of interest measurements was adapted and expanded to accommodate typical clinical PC measurements and the region-of-interest analysis used for such measurements. This included the dependency between cardiac phases that arises due to retrospective cardiac gating used in such studies. The proposed method enables calculation of standard deviations of flow measurements without the need for repeated experiments or repeated reconstructions. The method was compared to repeated trials in phantom measurements and pseudo replica reconstructions of in vivo data. Three different flow protocols (free breathing and breath hold with various accelerations) were compared in terms of the confidence interval ranges caused by thermal noise in the measurement data.

**Results:**

Using the proposed method it was possible to accurately predict confidence intervals for flow measurements. The method was in good agreement with repeated measurements in phantom experiments and there was also good agreement with confidence intervals predicted by pseudo replica reconstructions in both phantom and in vivo data. The proposed method was used to demonstrate that the variation in cardiac output caused by thermal noise is on the order of 1% in clinically used free breathing protocols, and on the order of 3-5% in breath-hold protocols with higher parallel imaging factors.

**Conclusions:**

It is possible to calculate confidence intervals for Cartesian PC contrast flow measurements directly without the need for time-consuming pseudo replica reconstructions.

## Background

It has long been recognized that nuclear magnetic resonance signals can be used to measure blood flow rates [1]. Since the introduction of the Phase Contrast (PC) technique [2,3], it has been used widely for clinical blood flow measurements. Phase contrast measurements are commonly used in several cardiovascular magnetic resonance (CMR) protocols [4,5] and they play a critical role in the evaluation of congenital heart disease [6,7]. Phase contrast flow measurements exhibit variability when repeated in the same subject. This variability can either be caused by physiological changes, i.e. real changes in flow, or the measurements may be influenced by errors. There are in principle two types of errors that affect either the accuracy (bias) or precision (noise). In general the accuracy is affected by experimental imperfections that cause the flow measurements to be biased. Examples of such experimental imperfections include eddy current effects and gradient imperfections. The accuracy problems caused by experimental imperfections are to some extend deterministic in nature and they can potentially be corrected if suitable calibration or correction data can be measured [8-10]. Other measurement imperfections such as errors caused by vessel segmentation are not necessarily deterministic and can be hard to correct. The noise level and how this noise propagates through the measurement and analysis determine the lower bound of the precision. Like all CMR measurements, PC studies are affected by thermal noise. This thermal noise propagates from the individual k-space samples through the reconstruction to the image pixels and finally through the analysis to the volume flow measurements calculated using region of interest (ROI) measurements. As a result, all PC derived measurements such as flow curves, stroke volumes, or Qp:Qs (ratio of pulmonary to systemic flow) have uncertainty (or confidence interval) associated with them caused by thermal noise. This confidence interval is influenced by a number of parameters such as magnetic field strength, field of view, spatial resolution, actual flow rates, velocity sensitivity settings, vessel sizes, contrast agents, receive coil configuration, parallel imaging, reconstruction algorithm, etc. Some of these parameters remain fixed from patient to patient but others change regularly and may affect the confidence interval (or precision) of flow measurements in ways that are less than intuitive. This makes it challenging to calculate the confidence intervals or the precision for flow measurements.

There are, however, some situations where it could be important to know the precision of a flow measurement. One example is a study comparing methods for correction of biases caused by experimental imperfections [8-10]. An important gauge of the effectiveness of such methods is the magnitude of the flow curve correction relative to the perturbation of the flow curve that one could expect due to noise alone. Another example is the study of subtle changes in flow curves due to some physiological change, e.g. breath-holding or Valsalva maneuver. In such experiments it is important to be able to examine if observed changes in flow could reasonably be explained by noise.

The noise in PC measurements is determined by the phase noise in the measurement scaled by the velocity sensitivity (VENC) and the area of the vessel of interest. In general the phase noise is related to the Signal to Noise Ratio (SNR) in each pixel [3,11]. The SNR in each pixel can usually be determined directly during the reconstruction for most Cartesian reconstructions [12,13], but since PC flow analysis involves multiple pixels in an ROI, it is necessary to know the standard deviation of some linear combination of the pixel phases. The ROI standard deviation will also depend on how the noise in the pixels is correlated. In an experiment where the pixels are independent, it is trivial to determine the standard deviations associated with an ROI measurement, but that is rarely the case due to raw data filtering, anisotropic pixel sizes, parallel imaging, etc.

One reliable way of determining the confidence interval for flow measurements (and other experiments) is the pseudo-replica method [14]. This method works by generating multiple reconstruction replicas. Each replica is generated by adding noise to the original raw data with the same noise statistics (levels, distribution, and correlation) as the original experiment and repeating the reconstruction. The confidence intervals for the flow measurements can then be obtained as the standard deviation of the analysis results from all reconstructions. Although this method is completely general and works for any kind of flow measurement, it is computationally intensive and time consuming.

We have recently described a direct method for calculating the standard deviation of any linear combination of pixel values (magnitude or phase) in Cartesian MRI reconstructions [15]. This method does not rely on repeated reconstructions and can be applied to phase measurements as long as the k-space sampling pattern and the reconstruction process are known. The purpose of this work is to adapt this method for direct calculation of confidence intervals on phase contrast flow measurements. The method will be used to calculate error bars on typical patient flow curves and to determine confidence intervals for derived measures such as cardiac output and Qp:Qs.

## Methods

### Theory

Phase contrast flow analysis consists of a segmentation of the vessel of interest and a summation of the signal phase (phase difference) within the segmented ROI. In order to estimate the standard deviation and thus confidence intervals on flow measurements, a previously published technique for ROI standard deviation calculation [15] was adapted for phase contrast flow imaging. The basic concepts of this technique are reviewed here before discussing the specific adaptation to flow quantification.

An ROI measurement can be thought of as a linear combination of the image pixel values. If **ρ** is a column vector containing all the pixels of the image and **m** is a column vector containing linear weighting coefficients (typically 0 or 1) corresponding to each pixel, then the result *r* (scalar) of an ROI measurement can be written as:

(1) $$r=m^{H}\;\rho$$

The variance of *r* is

(2) $$\sigma_{r}^{2}=m^{H}\;\Sigma_{\rho }m$$

Where **Σ**_**ρ**_ is the covariance matrix of the pixel noise. It is challenging to calculate $\sigma_{r}^{2}$ because **Σ**_**ρ**_ is a very large matrix, which in the general imaging case has a fairly complicated structure dictated by specific imaging parameters such as resolution, raw data filtering, parallel imaging, etc. It is not practical to form **Σ**_**ρ**_ explicitly, but it is possible to obtain a useful expression for **Σ**_**ρ**_ by exploiting the fact that **Σ**_**ρ**_ is related to the image reconstruction process in the following way:

(3) $$\Sigma_{\rho }\;=F\;\Sigma_{k}F^{H}$$

Where **Σ**_**k**_ is the covariance matrix of the k-space sample noise and **F** is the matrix that described the image reconstruction process. It is straightforward to ensure that **Σ**_**k**_ is diagonal since the noise in k-space is not generally correlated between k-space locations. With standard procedures for noise pre-whitening [12], **Σ**_**k**_ is simply the identity matrix. In the Cartesian imaging, a multiplication **F** or **F**^H^ can be expressed in terms of Fourier transforms and vector-vector multiplications. By combining equations (2) and (3), an expression for the variance of an ROI is obtained:

(4) $$\sigma_{r}^{2}=m^{H}F\;\Sigma_{k}F^{H}m$$

In the case of PC flow quantification, it is the phase image, which is being analyzed. As described in [15], equation (4) can be modified to deal with this non-linear extraction of phase information:

(5) $$\sigma_{\text{phase}\;\text{roi}}^{2}=m^{H}\theta^{H}\mathrm{MF}\;\Sigma_{k}F^{H}M\theta m$$

Where **θ** is a diagonal matrix with complex numbers along the diagonal, with magnitude 1 and phase equal to the image phase where the image has a well-defined phase (there is some signal), and zero otherwise. **M** is a diagonal matrix with an estimate 1/|ρ| (the reciprocal of the signal magnitude) along the diagonal. Equation (5) uses the approximation that the standard deviation of the phase is proportional to the reciprocal of the SNR. The PC technique uses the phase subtraction of two acquisitions with different flow sensitivities to eliminate background phase. This is easily captured in equation (5) by considering the reconstruction of both acquisitions jointly. Specifically, the **m** vector has twice the number of elements as there are image pixels and the ROI used in flow analysis is represented twice (once for each acquisition) using either ‘1’ or ‘-1’ corresponding to how a given acquisition is used in the phase subtraction. Using the expression in equation (5) it is possible to estimate variance of an ROI measurement without the need for pseudo replica reconstructions. The operations consist of only Fourier transforms and vector-vector multiplications and can be evaluated efficiently. In the case of phase contrast measurements, it is necessary to scale the phase standard deviation obtained using equation (5) by the velocity encoding sensitivity (VENC) and the ROI area to obtain the standard deviation of the flow measurements.

Phase contrast flow measurements are most often done with retrospective cardiac gating [16]. As a result each line in a given k-space frame (a specific cardiac phase) is formed through some interpolation process using k-space lines that were acquired at approximately the time of the cardiac phase. Since some lines in k-space may have more contributing lines than others, the k-space variance will vary from line to line. More explicitly, **Σ**_**k**_ is still a diagonal matrix but it is not identity, the variance for each k-space line can be expressed as:

(6) $$\sigma_{k}^{2}=\frac{\sum_{i=1}^{N_{w}}w_{i}^{2}}{\sum_{i=1}^{N_{w}}w_{i}}$$

where *w*_*i*_ are the interpolation weights assigned to each line of k-space contributing to a given k-space location and *N*_*w*_ is the total number of lines contributing to a given location.

The objective of most phase contrast studies is to obtain not only the instantaneous frame by frame flow rates but also derived measures such as cardiac output Q (the time integral of the flow curve) and Qp:Qs. If the cardiac phases are assumed to be independent, the variance of the volume flow Q is simply the sum of the variances of each individual phase. However, the retrospective gating involves temporal interpolation, which is a convolution of the k-space data over time with some kernel. Assuming the same kernel for every cardiac phase, it is possible to obtain a single number for the frame independence based on the noise equivalent bandwidth of the kernel. If the convolution kernel is a pre-calculated kernel (lookup table) with *N*_*s*_ samples then the frame independence factor is calculated as:

(7) $$f=\sqrt{\frac{\sum_{i=1}^{N_{s}}{w_{i}}^{2}\;\text{dw}}{{\left(\sum_{i=1}^{N_{s}}\;w_{i}\text{dw}\right)}^{2}}}$$

where *dw* is the kernel sample width, and *w*_*i*_ are the kernel samples. Using the factor *f* it is possible to obtain an estimate of the standard deviation of stroke volume (or any other volume obtained by integrating cardiac phases) using the equation:

(8) $$\sigma_{\text{volume}}=\sqrt{\sum_{i=1}^{N_{p}}\sigma_{i}^{2}/f^{2}}$$

where *N*_*p*_ is the number of cardiac phases in the volume estimate and $\sigma_{i}^{2}$ is the variance of the instantaneous flow at each cardiac phase.

For ratio measurements such as regurgitant fraction and Qp:Qs, the standard deviation is not well defined if the confidence interval of the denominator includes zero. In general, that is not the case for the typical flow studies and in the approach used in this paper, the following approximation for ratio measurements is used:

(9) $$\sigma_{A/B}=\frac{A}{B}\sqrt{{\left(\sigma_{A}/A\right)}^{2}+{\left(\sigma_{B}/B\right)}^{2}}$$

### Reconstruction and analysis

**Figure 1:**
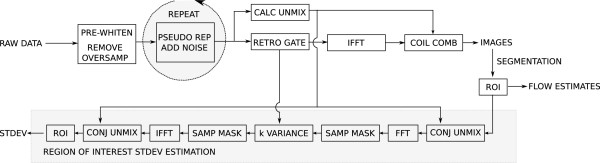
**Simplified outline of image reconstruction and analysis pipeline.** The data is first subjected to noise pre-whitening and oversampling in the readout direction is removed. After pre-whitening, retrospective gating is used to bin the data into cardiac phases. Data are then Fourier transformed to image space and the multi-channel data is combined with unmixing coefficients that are calculated from GRAPPA calibration data. The reconstruction can be repeated multiple times with added random noise using the optional pseudo replica module. After image reconstruction, ROIs are defined on the images and based on the ROI geometry, the sampling pattern, and unmixing coefficients the standard deviation of the ROI phase sum is calculated according to equation (5).

The method presented in this study does not rely on a particular reconstruction algorithm as long as the expression in equation (5) can be evaluated efficiently as is the case with most Cartesian reconstruction algorithms. The particular reconstruction and analysis pipeline used to process the data in this study is outlined in Figure 1. The acquired data was pre-whitened [12] based on noise data from a calibration acquisition. After noise pre-whitening, readout oversampling was removed by Fourier transform to image space and field of view truncation. The data was then transformed back to k-space. At this point in the reconstruction the data was assumed to have noise variance equal to 1 in all receive channels and the noise was assumed to be uncorrelated between receive channels and k-space locations. The data was assigned to cardiac phases using a linear interpolator (Bartlett window) for each k-space line using the measured RR interval for each heartbeat of the segmented acquisition. After cardiac phase interpolation, the data was Fourier transformed to image space and the data from multiple receive coils were combined using a set of phased array combiner coefficients. These coefficients were estimated using GRAPPA calibration data. External calibration data was used in this study. The calibration data was used to calculate k-space convolution kernels. These kernels were zero-padded and transformed to image space where a coil sensitivity estimate [17] was used to combine the image space coefficients to a single set of unmixing coefficients. After coil combination the reconstruction result was comprised of two complex images for each cardiac phase corresponding to the two velocity encoding measurements.

The reconstructed images were fed into an in-house developed analysis tool (Matlab, The Mathworks, Natick, Massachusetts, U.S.A.) that enabled segmentation of vessels such that the ROIs needed to form **m** in equation (5) could be defined. In practice, **m** was a vector with twice as many elements as there were image pixels. Each element had a value of either 1, -1, or 0 corresponding to pixels that were added, subtracted, or not included in the ROI. The image analysis also included information about the unmixing coefficients and the k-space variances, **Σ**_**k**_, which were estimated during the retrospective cardiac gating procedure. Based on these inputs, the standard deviation of the instantaneous flow in a given cardiac phase was estimated using equation (5). The analysis tool also included a concomitant field correction step [18] and a background phase correction. Background pixels were identified based on the estimated phase variation as determined by the SNR of the individual pixels. All pixels with a lower phase variance than predicted by the SNR and with an SNR higher than 10 were designated background pixels and a plane (1^st^ order correction) was fit through the phase of these background pixels to remove background phase.

In order to validate the proposed method, multiple pseudo replica reconstructions were also generated by repeated reconstructions with added white noise. These repeated reconstructions were also analyzed using the image analysis tool and the standard deviation of the flow values across the pseudo replicas was considered an independent estimate of the standard deviation.

### Phantom measurements

As an initial validation of the method, a static phantom was studied using a gradient echo phase contrast sequence with the following parameters. Matrix size: 128x128, Field of View: 240×240 mm, Flip Angle: 20, TE: 2.79, TR: 5.07, parallel imaging factor: 2, reconstructed cardiac phases: 10. In this and all subsequent phantom and in vivo experiment we chose a velocity encoding sensitivity (VENC) of 200 cm/s. The VENC was chosen to avoid aliasing in the in vivo experiments and kept constant to enable a more direct comparison of the confidence intervals between measurements. A simulated ECG signal was used to assign cardiac phase timing to the acquired data. The acquisition was repeated 100 times to provide a true repeated experiment validation and additionally 100 pseudo replica reconstructions were generated based on the first of these repeated experiments. Two different sizes of ROIs were segmented on the static phantom and flow analysis was performed as outlined in Figure 1. The ROIs were circular with an area of 56 pixels for the small ROI and 347 pixels for the large ROI.

A CardioFlow 5000 (Shelley Medical Imaging Technologies, London, Ontario, Canada) pulsatile flow pump was used to generate a femoral flow curve with an nominal net flow rate of 2400 ml/s. Tubing with the pulsatile flow was placed next to a static phantom in the scanner to provide adequate signal for frequency adjustment, etc. The tube was imaged with a segmented gradient echo phase contrast sequence with the following parameters. Field of view: 240×180mm, matrix size: 192×144, phase resolution: 50%, flip angle: 20 degrees, VENC: 200 cm/s, TE: 3.11 ms, TR: 5.16 ms, parallel imaging acceleration factor: 4, acquired cardiac phases: 23, reconstruction cardiac phases: 30. The flow pump provided the ECG gating signal needed for retrospective gating. The measurement was repeated 100 times and 100 pseudo replica reconstructions were generated based on the first of the repeated measurements. Standard deviations for instantaneous flow values and cardiac output were generated using both repeated experiments and pseudo replicas. Since this acquisition used a significantly reduced phase resolution it was also used to compare the standard deviations for cardiac output obtained using the assumption that all pixels in the reconstructed images were independent. The number of pixels within the ROI covering the tube was 90.

### In vivo measurements

The proposed method was used to compare three different flow protocols in six (N = 6) patients studied at Children’s National Medical Center, Washington, DC. Written informed consent was obtained for all studies and the local institutional review board approved the study protocol. The pertinent parameters of the flow protocols are outlined in Table 1. A standard free breathing protocol with multiple averages was acquired along with two protocols with higher parallel imaging acceleration factors. The acquisitions with higher acceleration factor were acquired in a breath-hold using only a single signal average. Flow measurements were obtained in the ascending aorta (Qs) and in the main pulmonary artery (Qp). In all acquisitions, confidence intervals for instantaneous flow measurements (for each cardiac phase) and cardiac output were calculated using the proposed method and estimated using the pseudo replica method. Confidence intervals were also calculated for Qp:Qs ratios. The ROI sizes varied from 50 to 200 pixels in the in vivo experiments.

**Table 1:** In vivo phase contrast sequence parameters

	**Free breathing, R = 2**	**Breath hold, R = 3**	**Breath hold, R = 4**
	**(FB)**	**(BH3)**	**(BH4)**
Matrix	240x180-240	192x144-192	192x144-192
Field of view	360x240-360	360x240-360	360x240-360
Parallel imaging	2	3	4
Flip angle	20	20	20
TE	2.6-2.8 ms	2.5-2.7 ms	2.5-2.7
TR	5.0-5.2 ms	4.5-4.7 ms	4.5-4.7 ms
VENC	200	200	200
Averages	3	1	1
Acquired phases	30-40	23-26	20-25
Reconstructed phases	30	30	30

Flow protocols used for in vivo comparison. R = acceleration factor.

## Results and discussion

**Figure 2:**
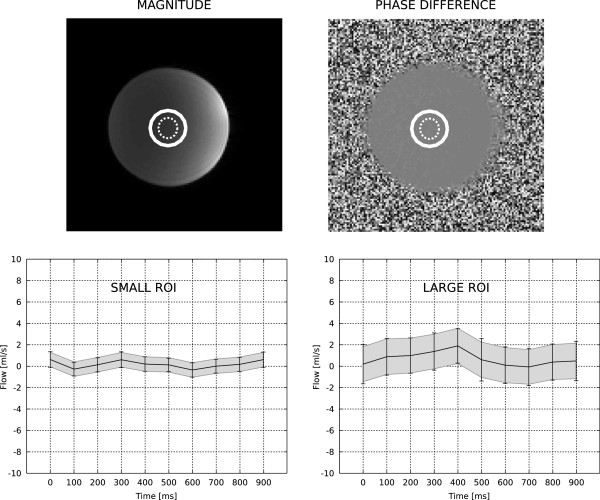
**A comparison of the proposed method and a true repeated measures experiment in a static phantom.** The static phantom and phase difference image is depicted in the top part of the figure and the curves (bottom) show the instantaneous flow rates calculated for the ten simulated cardiac phases. The gray shaded area around the flow curve indicates the 95% (1.96 * σ) confidence interval (CI) predicted by the proposed method and the error bars indicate the 95% CI measured from 100 repeated experiments. Analysis was performed for two different ROI sizes; small ROI is depicted with a dotted line on the images and the large ROI is depicted with a solid line.

**Figure 3:**
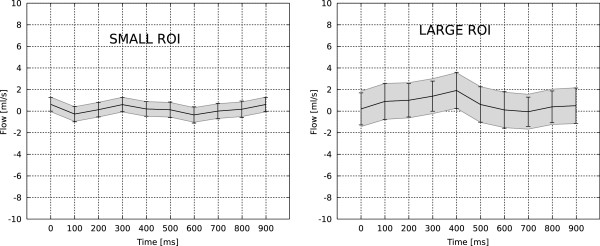
**A comparison of the proposed method and a pseudo replica experiment using a static phantom.** An illustration of the static phantom and the ROI placement can be found in Figure 2. The gray shaded area around the flow curve indicates the 95% (1.96 * σ) confidence interval (CI) predicted by the proposed method and the error bars indicate the 95% CI measured from 100 pseudo replica reconstructions.

All flow measurement were analyzed with the proposed method. The evaluation of equation (5) for all cardiac phases and associated calculations for net flow measurements took on the order of 5 seconds for a typical flow measurement. The results of the static phantom experiment are depicted in Figures 2 and 3. Figure 2 shows the comparison of the proposed method and true repeated experiments. There is good agreement between the 95% confidence interval (CI) predicted using equation (5) and the repeated experiments. Similarly, Figure 3 demonstrates the good agreement between the CIs predicted by the proposed method and pseudo replica experiments. Note that as the ROI increases in size, the uncertainty of the flow measurement increases, since the instantaneous flow value is obtained by integration of all the pixels within the ROI. The phantom experiment constitutes a very basic validation of both the proposed method and the pseudo replica analysis setup used in this paper.

**Figure 4:**
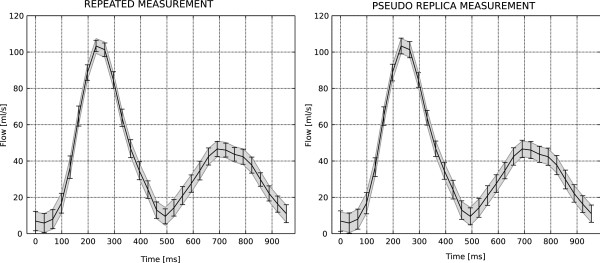
**A comparison of confidence intervals predicted by the proposed method and both repeated experiments (left) and pseudo replica method (right) in a pulsatile flow phantom.** The gray shaded area around the flow curves indicates the 95% (1.96 * σ) confidence interval (CI) predicted by the proposed method and the error bars indicate the 95% CI measured from 100 repeated experiments on the left and 100 pseudo replica reconstructions on the right. The measured net flow rate was 2288 ml/s and the net flow 95% CIs were 87.44 ml/min, 90.94 ml/min, and 90.87 ml/min for the proposed method, the repeated experiment, and the pseudo replica method respectively.

Figure 4 depicts the flow curves from the pulsatile flow phantom experiment. The left panel compares the predicted CIs to the CIs obtained by repeating the experiment 100 times. The right panel compared the proposed method to the pseudo replica technique. Again, there is good agreement between the predicted confidence intervals. The measured net flow was 2288 ml/min and the 95% CIs were 87.44 ml/min, 90.94 ml/min, and 90.87 ml/min for the proposed method, the repeated experiment, and the pseudo replica method respectively. The CI of the net flow obtained by assuming that the pixels were independent was 60.72 ml/min for this experiment. This is differs from the CI obtained with the proposed method by approximately  as expected given the 50% phase resolution used in this experiment. It should be noted that the calculation using the assumption of independent pixels still uses large parts of the presented framework to obtain pixel-wise standard deviations of the phase and it also uses the correction for correlation between cardiac phases as outlined in equations (7) and (8).

**Figure 5:**
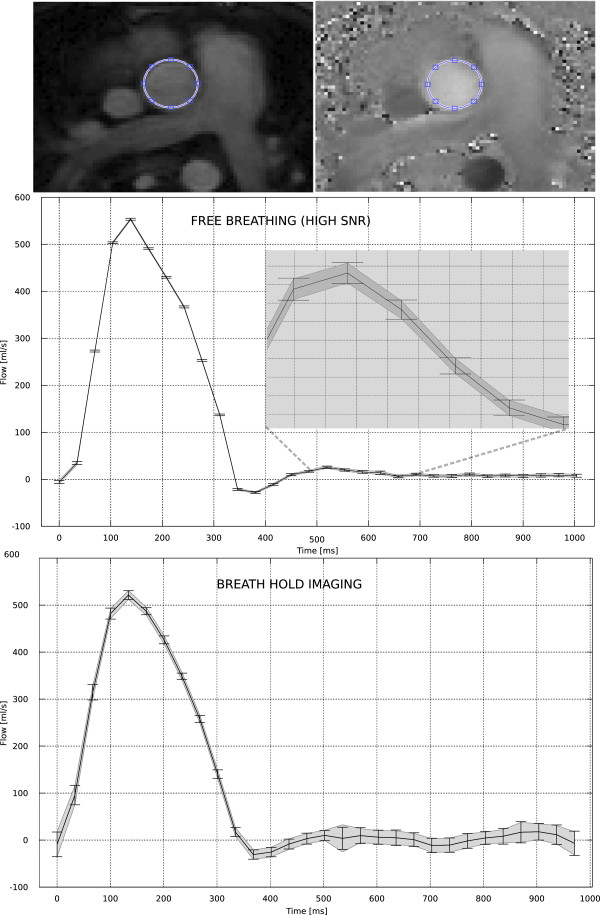
**Example patient flow curves from an aortic flow measurement.** Example images are seen (top) with indication of ROI placement. The two curves are from a free-breathing (multiple averages), high SNR acquisition in the middle and from a parallel imaging rate 4, breath-hold acquisition on the bottom. The gray shaded area around the flow curves indicate the 95% (1.96 * σ) confidence interval (CI) predicted by the proposed method and the error bars indicate the 95% CI measured from 100 pseudo replica reconstructions. The free breathing acquisition has very low noise and very tight CIs and a zoom of the curve has been inserted to illustrate the agreement between the proposed method and the pseudo replica technique.

**Figure 6:**
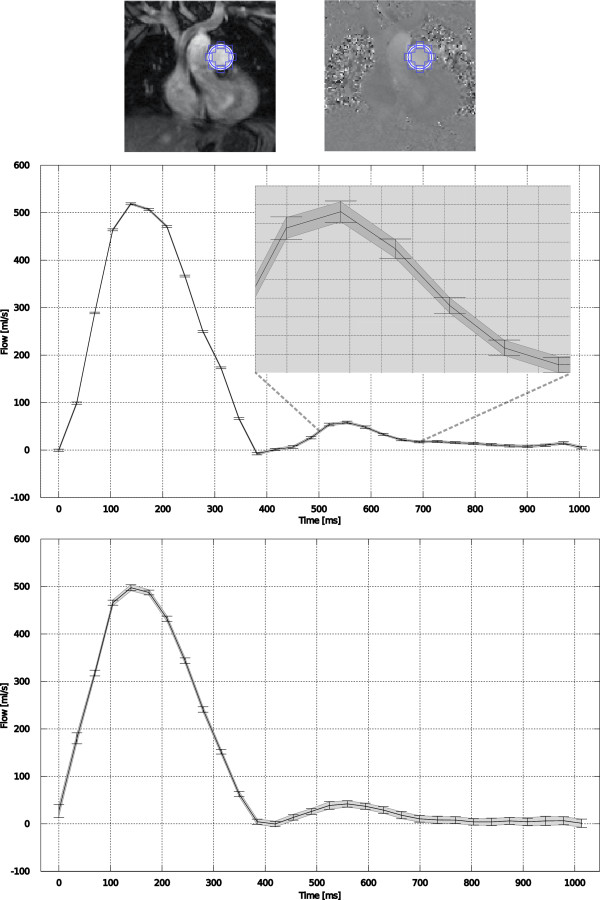
**Example patient flow curves from a pulmonary artery flow measurement.** Example images are seen (top) with indication of ROI placement. The two curves are from a free-breathing (multiple averages), high SNR acquisition in the middle and from a parallel imaging rate 4, breath-hold acquisition on the bottom. The gray shaded area around the flow curves indicate the 95% (1.96 * σ) confidence interval (CI) predicted by the proposed method and the error bars indicate the 95% CI measured from 100 pseudo replica reconstructions. The free breathing acquisition has very low noise and very tight CIs and a zoom of the curve has been inserted to illustrate the agreement between the proposed method and the pseudo replica technique.

In the pulsatile flow phantom experiment, the measured net flow rate differed from the nominal flow rate of 2400 ml/min. This deviation was more than can reasonably be attributed to noise given the calculated confidence intervals and consequently it can be concluded that either a) the measurement was biased (e.g. by some uncorrected background phase) or b) the pulsatile flow phantom was not appropriately calibrated. This particular experiment does not provide the data required for determining the exact cause of the deviation, but it does provide the confidence to say that there is a statistically significant deviation from the expected net flow rate.In vivo datasets were obtained with three different protocols in six patients. In one patient, the pulmonary flow measurement acquired with parallel imaging rate 4 was corrupted by parallel imaging artifacts and could not be analyzed. All other datasets were analyzed. Example in vivo flow curves can be seen in Figure 5 (aortic flow) and Figure 6 (pulmonary flow). In these in vivo experiments, it is only possible to compare the proposed method to the pseudo replica technique, since repeated experiments in vivo would include physiological variation in addition to the variation caused by thermal noise. The flow curves demonstrate that there is good agreement between the CIs predicted by the proposed method (gray shaded area) and the pseudo replica technique (error bars), indicating that the technique can be applied to in vivo data. The average (of all subjects) systemic cardiac output measured with the three flow protocols was 5230 ml/min, 5161 ml/min, and 5070 ml/min for the free breathing, rate 3, and rate 4 protocols respectively. In the pulmonary artery, the three protocols measured 5501 ml/min, 5573 ml/min, and 5376 ml/min, and the mean resulting Qp:Qs ratios obtained with the three protocols were 1.05, 1.09, and 1.02.

**Figure 7:**
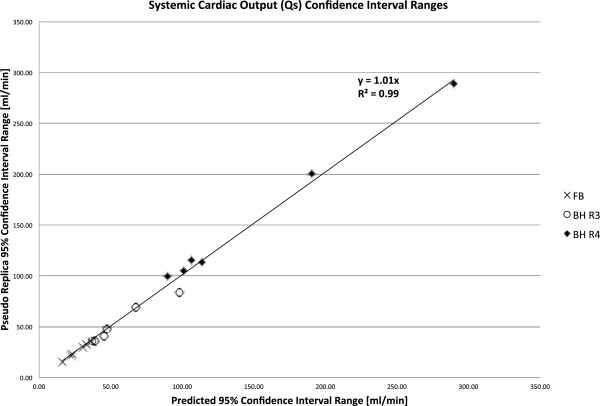
**Comparison of confidence intervals (CIs) on systemic flow predicted with the proposed method and the pseudo replica technique.** The graph includes data from three protocols. FB: Free Breathing, 3 Averages, BH R3: Breath Hold, Rate 3, BH R4: Breath Hold, Rate 4.

**Figure 8:**
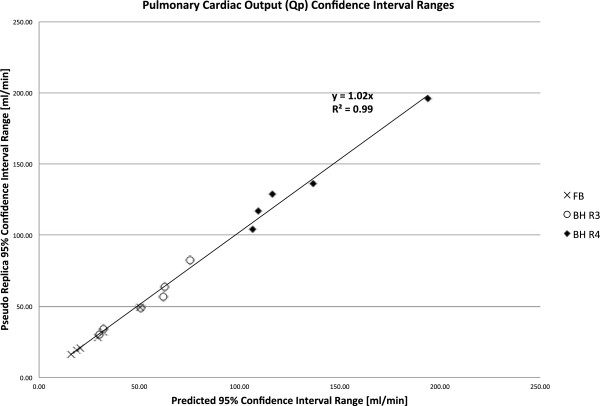
**Comparison of confidence intervals (CIs) on pulmonary flow predicted with the proposed method and the pseudo replica technique.** The graph includes data from three protocols. FB: Free Breathing, 3 Averages, BH R3: Breath Hold, Rate 3, BH R4: Breath Hold, Rate 4.

**Figure 9:**
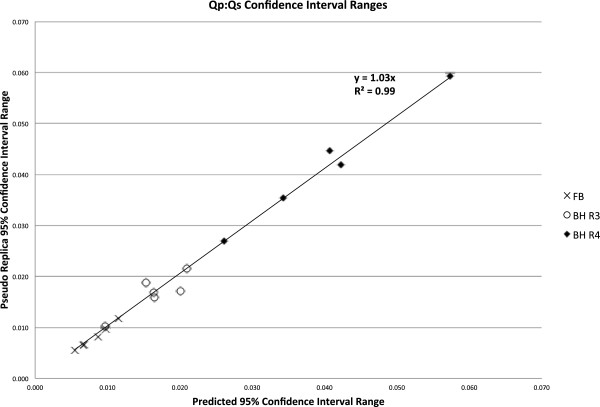
**Comparison of confidence intervals (CIs) on Qp:Qs ratios predicted with the proposed method and the pseudo replica technique.** The graph includes data from three protocols. FB: Free Breathing, 3 Averages, BH R3: Breath Hold, Rate 3, BH R4: Breath Hold, Rate 4.

The purpose of this study was to compare the CIs predicted by the proposed method and the pseudo replica method. Figures 7, 8, and 9 demonstrate the range of confidence intervals for Qs, Qp, and Qp:Qs ratios respectively. The CI ranges obtained with the pseudo replica method are plotted against the CI ranges predicted by the proposed method. There is good agreement between the proposed method and the pseudo replica method. The correlation is excellent (R^2^ > 0.99) and the slope of the correlation curve is close to 1 for all three compared parameters. The curves also demonstrate the ranges of these confidence intervals. For the free breathing protocol with multiple averages, thermal noise contributed very little to any variation in flow measurements (on the order of 1%). In the higher acceleration breath-held cases thermal noise contributes more to the variation, but it is still generally below 5%. Similar conclusions can be drawn regarding the Qp:Qs ratios. It is well known that variations in flow measurement (when repeated in the same patient) are higher than this. With the method presented here it is possible to separate the part of the variation caused by thermal noise from other sources of variation, i.e. physiological variation and biases. The value of the proposed method lies in this ability to separate the sources of variation. This is valuable when comparing two different sequences for measuring flow in the same location. If there is a difference between two methods, it is important to have a way to determine if this difference could reasonably be explained by noise alone. Another important application is the study physiological changes in the same subject over time or in response to some stimulus or challenge (e.g. breath-holding). In fact, if such an experiment is constructed where any biases (sequence choices, etc.) are kept constant, the confidence intervals calculated by presented method would represent the lower bound of the variation and it would be possible to obtain estimates of the variation caused by physiological changes. It was, however, not the purpose of this study to characterize such physiological changes.

The experiments this paper were focused on 2D flow measurements, but the method is directly applicable to 3D imaging as well, i.e. it could be used to analyze so-called 4D flow measurements. The evaluation of equation (5) requires two Fourier transforms of the datasets and while the computation time is negligible in the 2D case, some optimization of the code and workflow would be needed to enable a seamless workflow for 3D imaging. It should also be noted that 4D flow studies are often conducted to explore more advanced flow analyses such as streamline visualization or wall shear rate analysis. The presented method does not enable confidence calculation for such measurements. However, the general approach that has been employed here could be expanded to analyze such measurements.

Most flow measurement analyses includes some form of background correction using static phantoms or background fitting [8,19]. These background correction techniques also have thermal noise contributions. Specifically, background fitting could be very susceptible to pixel noise if a limited number of pixels are used and static phantom acquisitions suffer from the same thermal noise propagation as characterized in this study. If the static phantom measurement is performed with the same protocol as used in the in vivo study and it is assumed to have the same SNR as the in vivo measurement, it is reasonable to assume that phantom correction increases the confidence intervals by . Specifically, if the phase variance is the same in both the in vivo measurement and the static phantom measurement, the variance of the phase subtraction will double and thus the standard deviation will increase by . The precision loss due to background fitting will depend on the fitting technique and the number of pixels used. This study did not attempt to characterize the noise properties of possible background correction options. The analysis conducted here assumes that the background correction terms were constant from measurement to measurement.

The advantage of the proposed method over the pseudo replica technique is that it can be evaluated efficiently without the need for repeated reconstruction. As such it would be suitable for direct integration into a flow analysis software package where the confidence intervals could be evaluated on the fly. The method does, however, have some limitations. In the present form, it only works for Cartesian reconstructions where the reconstruction process is known. In the special case of non-Cartesian imaging without parallel imaging acceleration, the method is also directly applicable, but for non-Cartesian parallel imaging reconstructions or in situations where the reconstruction implementation is unknown, the pseudo replica method would be useable albeit at the expense of a considerable increase in evaluation time that would be prohibitive for integration with an analysis software package.

## Conclusion

We have demonstrated a method for direct calculation of the variation caused by thermal noise in phase contrast flow measurements, which limits the precision. The method can be used to calculate confidence intervals on both instantaneous flow for a given cardiac phase and derived measures such as cardiac output and Qp:Qs ratios.

## Abbreviations

CI: Confidence interval; CMR: Cardiovascular magnetic resonance; PC: Phase contrast; Qp: Pulmonary flow; Qs: Systemic flow; Qp:Qs: Ratio of pulmonary to systemic flow; ROI: Region of interest; SNR: Signal to noise ratio.

## Competing interests

The authors declare that they have no competing interests.

## Authors’ contributions

MSH: Conceived the study, developed the theoretical foundation for the presented method, provided a practical implementation of the method including all software used in the study, analyzed data, wrote manuscript. KO: Acquired patient data, identified reference data for method development, assisted with data analysis. LJO: Provided clinical context for the method development, acquired patient data, assisted with data analysis. RRC: Discussed clinical implications of method, acquired patient data, assisted with data analysis. SJI: Contributed to the development of the mathematical foundation of the method, provided feedback on data analysis, assisted with drafting of the manuscript. PK: Participated in conceiving the study, contributed to the development of the mathematical foundation of the algorithms used, helped draft the manuscript. All authors read and approved the final manuscript.
